# The Impact of Body Mass Index on the Clinicopathological and Prognostic Factors of Colorectal Cancer in Saudi Arabia

**DOI:** 10.7759/cureus.11789

**Published:** 2020-11-30

**Authors:** Saleh M Aldaqal, Abdulqader A Maqbul, Ahmed A Alhammad, Aseel S Alghamdi, Bandar A Alharbi, Meshal T Alharbi, Omar M Alhazmi, Yasir O Zaylaee

**Affiliations:** 1 General Surgery, King Abdulaziz University Faculty of Medicine, Jeddah, SAU

**Keywords:** colorectal cancer, obesity, body mass index, prognosis, clinicopathology, bmi

## Abstract

Background

Obesity is a known risk factor of colorectal cancer (CRC); however, the relationship between obesity and clinicopathologic characteristics and prognosis of CRC remains unclear. This study aimed to investigate the relationship between body mass index (BMI) and clinicopathological and prognostic factors of CRC in Saudi Arabia.

Method

This was a retrospective cross-sectional study of patients with CRC diagnosed between 2014 and 2018 at King Abdulaziz University Hospital in Jeddah, Saudi Arabia. BMI was calculated by dividing the patient’s weight in kilograms by height in meter squared and was classified according to the World Health Organization criteria. Statistical tests, including analysis of variance and chi-square tests, were used to investigate the relationship of each BMI category with clinicopathologic (histological type, degree of differentiation, tumor location, and medical comorbidities) and prognostic variables (TNM stage, lymph nodes involvement, and lymph nodes yield).

Results

Of 233 patients who were included, 60.1% were male and 39.9% were female patients, with a mean age (standard deviation) of 58.8 ± 13.7 (range: 26-99) years. The median BMI was 26.5 kg/m^2^. Overall, 3%, 34.3%, 33.0%, and 29.6% patients were classified as underweight, normal weight, overweight, and obese, respectively. Furthermore, 57.1% (4/7), 39.2% (31/80), 38.7% (29/77), and 25.8% (17/69) of underweight, normal, overweight, and obese patients had Stage IV disease (p = 0.20). Of 16 patients with transverse colon cancer, 8 (50%) were obese (p = 0.38), and 1 (6%), 5 (31%), and 2 (13%) were underweight, normal weight, and overweight, respectively.

Conclusion

Underweight patients are more likely to present with metastatic CRC, while obese patients are more likely to present at earlier stages, although the difference was not statistically significant. BMI is not related to lymph node yield, histological type, or the degree of differentiation.

## Introduction

Colorectal cancer (CRC) is the third most common malignancy and the fourth most common cause of cancer deaths worldwide [1]. In Saudi Arabia, CRC was the first and third most common malignancy among men and women, respectively, in 2012 [2]. Several factors such as smoking, high consumption of red and processed meat, history of inflammatory bowel disease, personal or family history of CRC, and obesity have been found to be associated with a higher risk of CRC [3].

Obesity is a growing public health concern worldwide. In 2013, obesity was prevalent in approximately 30% of adult men and almost 15% of adult women [4]. Obesity is a major modifiable contributing factor of the incidence of various types of cancers, including colon cancer, which is considered to be the second most common obesity-related cancer [5]. Increasing evidence supports the relationship between body mass index (BMI) and the increased risk of colorectal adenoma [6] and CRC [7]. Additionally, a study found that overweight and obesity in adolescence are associated with the development of colon cancer in later life [8]. A systematic review and meta-analysis of prospective observational studies showed that a 5-kg/m^2^ increase in BMI was significantly associated with the development of colon cancer [7].

In addition to predisposing individuals to CRC, patients with obesity and CRC are found to have lower survival rates than those in other BMI groups [9,10]. A meta-analysis that compared outcomes between obese and non-obese patients undergoing surgery for CRC demonstrated a higher probability of lymph node metastases, lower lymph node yield, and more challenging resections in obese patients [11]. However, a recent study also found that overweight and obese patients were less likely to have advanced stages of CRC and had higher overall and cancer-specific survival rates [12]. Additionally, overweight but not obese patients with CRC had better overall prognosis than those in other BMI categories [13]. Based on these conflicting findings, the relationship between BMI and CRC outcomes remains unclear to date. As such, several studies have investigated whether obese patients have a higher risk of developing CRC within certain subsites of the colon or rectum compared with normal-weight patients. However, the results have been inconsistent [9,14].

To the best of our knowledge, no previous studies in Saudi Arabia have investigated clinicopathological and prognostic factors of CRC according to different BMI groups. Thus, this study aimed to examine the relationship of BMI with clinicopathological and prognostic factors of patients with CRC in Jeddah, Saudi Arabia.

## Materials and methods

Study design and patients

This single-center retrospective study was approved by the institutional ethics committee of King Abdulaziz University Hospital (KAUH) and obtaining informed consent was waived.

We evaluated patients who were diagnosed with CRC between 2014 and 2018 and who underwent surgery at KAUH (n = 1,064), a tertiary healthcare center. Patients with confirmed CRC via colonoscopy were eligible. The exclusion criterion was missing data on weight, height, and tumor stage. A total of 233 patients were enrolled in the study. Data were collected from medical records. Staging was performed according to the TNM classification 7th edition by The American Joint Committee on Cancer [15]. Height and weight were recorded at the time of presentation. BMI was calculated by dividing weight in kilograms with height in meters squared (BMI = kg/m^2^) and was classified according to the World Health Organization criteria as follows: underweight, <18.5 kg/m^2^; normal weight, 18.5-24.9 kg/m^2^; overweight, 25-29.9 kg/m^2^; and obese, >30 kg/m^2^ [16].

Statistical analysis

Data were entered in Microsoft Excel for analysis. Qualitative variables (sex; presence of comorbidities; tumor location; pathological characteristics including histological type, degree of differentiation, tumor extent [T], lymph node involvement [N], and presence of metastasis) are presented as frequencies. Meanwhile, quantitative variables (age at presentation, weight, height, and BMI) are presented as measures of central tendency (means and standard deviation (SD), or median and interquartile range (IQR)). A different bivariate chi-square test and analysis of variance (ANOVA) were used to analyze the relationship between two categorical variables, while ANOVA was used to analyze the relationship between categorical and numerical variables. All statistical analyses were performed using IBM SPSS Statistics for Windows, version 21 (IBM Corp., Armonk, NY). A p-value <0.05 was considered significant.

## Results

In total, 233 patients were included, of whom 60.1% and 39.9% were male and female patients, respectively. The mean age (standard deviation [SD]) at diagnosis was 58.8 ± 13.7 (range: 26-99) years. The mean [SD] height was 162.3 ± 8.8 cm, and the mean (SD) weight was 72.2 ± 16 kg. The median BMI for the overall cohort was 26.5 kg/m^2^, with male and female patients having mean BMIs of 25.9 kg/m^2^ and 28.3 kg/m^2^, respectively. Overall, 34.33% patients had normal BMI, 33.04% were overweight, 29.61% were obese, and 3% were underweight. Patient characteristics are shown in Table 1. Medical comorbidities were present in 35.6% patients, and the most frequent comorbidities were diabetes mellitus, hypertension, and ischemic heart disease.

**Table 1:** Patient characteristics according to BMI category BMI, body mass index; SD, standard deviation; IQR, interquartile range

	Underweight (BMI <18.5 , n = 7)	Normal weight (BMI 18.5–24.9, n = 80)	Overweight (BMI 25.0–29.9, n = 77)	Obese (BMI ≥30, n = 69)	Total (n = 233)
Age at diagnosis (mean, ± SD)	77.6 ± 9.38	58.1 ± 16.5	58.8 ± 12.0	58.5 ± 10.9	58.8 ± 13.7
Sex, n (%) Male Female	57.1 42.9	66.3 33.8	63.6 36.4	49.3 50.7	60.1 39.9
Weight, kg (mean, ± SD)	44.1 ± 6.1	59.5 ± 8.7	72.6 ± 8.2	89.4 ± 11.7	72.2 ± 16.0
Height, cm (mean, ± SD)	160.2 ± 8.8	162.8 ± 10.1	163.3 ± 8.4	160.8 ± 7.5	162.3 ± 8.8
BMI, kg/m^2^ (median, ± [IQR])	17.0 ± 0.8	22.8 ± 2.3	27.2 ± 2.5	34.1 ± 5.5	26.5 ± 7.7

In the bivariate analysis, the BMI category was not significantly associated with age at diagnosis (p = 0.111) or the presence of other medical comorbidities (p = 0.317). Adenocarcinoma was the most common histopathological type among all BMI groups, accounting for 85.7%, 85%, 87%, and 85.5% of all cases in the underweight, normal weight, overweight, and obese groups, respectively. The tumor characteristics with respect to the BMI groups are shown in Table 2. The sigmoid colon was the most frequent cancer site (27.8%), followed by the rectum (21%), and rectosigmoid (15%). The distribution of the tumor sites according to BMI categories is shown in Table 2.

**Table 2:** Tumor characteristics according to BMI category BMI, body mass index

	P-value	Underweight (BMI <18.5 kg/m^2^)	Normal weight (BMI 18.5-24.9 kg/m^2^)	Overweight (BMI 25-29.9 kg/m^2^)	Obese BMI (≥30 kg/m^2^)	Total
N (%)		7 (3.0)	80 (34.3)	77 (33.0)	69 (29.6)	233 (100)
Histological type	0.91					
Adenocarcinoma		6 (85.7)	68 (85.0)	67 (87.0)	59 (85.5)	200 (85.8)
Intramucosal carcinoma		0	0	0	1 (1.4)	1 (0.4)
Mucinous Adenocarcinoma		1 (14.3)	11 (13.8)	9 (11.7)	7 (10.1)	28 (12.0)
Neuroendocrine carcinoma		0	1 (1.3)	0	0	1 (0.4)
Signet ring cell adenocarcinoma		0	0	1(1.3)	1 (1.4)	2 (0.9)
Degree of differentiation	0.71					
Well-differentiated		2 (0.9)	6 (2.6)	10 (4.4)	8 (3.5)	26 (11.5)
Moderately differentiated		5 (2.2)	65 (28.6)	59 (26.0)	56 (24.7)	185 (81.5)
Poorly differentiated		0 (0)	6 (2.6)	5 (2.2)	5 (2.2)	16 (7.0)
TNM stage	0.20					
Stage I		1 (14.3)	11 (13.9)	7 (9.3)	13 (19.7)	32 (14.2)
Stage II		1 (14.3)	18 (22.8)	29 (38.7)	18 (27.3)	66 (29.2)
Stage III		1 (14.3)	19 (24.1)	10 (13.3)	17 (25.8)	47 (21.0)
Stage IV		4 (57.1)	31 (39.2)	29 (38.7)	17 (25.8)	81 (36.0)
Lymph node involvement	0.94					
N0		4 (57.1)	41 (51.3)	44 (57.1)	38 (55.1)	127 (54.5)
N1		2 (28.6)	21 (26.3)	20 (26.0)	15 (21.7)	58 (24.9)
N2		1 (14.3)	18 (22.5)	13 (16.9)	16 (23.2)	48 (20.6)
Lymph node yield	0.89					
Examined		Mean: 17.14 ± 3.44	Median: 14.00 ± 11.0	Median: 13.50 ± 14.3	Mean: 15.67 ± 7.30	Median: 14.00 ± 11.00
Affected		Median: 0.0 ± 1.0	Median: 0.0 ± 3.0	Median: 0.00 ± 2.0	Median 0.0 ± 3.0	Median: 0.0 ± 2.0
Tumor location	0.38					
Ascending colon		1 (14.3)	4 (5.0)	8 (10.4)	11 (15.9)	24 (10.3)
Cecum		1 (14.3)	12 (15.0)	10 (13.0)	4 (5.8)	27 (11.6)
Descending colon		0	5 (6.3)	6 (7.8)	6 (8.7)	17 (7.3)
Rectosigmoid		1 (14.3)	15 (18.8)	10 (13.0)	9 (13.0)	35 (15.0)
Rectum		1 (14.3)	22 (27.5)	15 (19.5)	11 (15.9)	49 (21.0)
Sigmoid		2 (28.6)	17 (21.3)	26 (33.8)	20 (29.0)	65 (27.9)
Transverse		1 (14.3)	5 (6.3)	2 (2.6)	8 (11.6)	16 (6.9)
Comorbidities	0.317					
Present		3 (3.6)	22 (26.5)	29 (34.9)	29 (34.9)	83 (38.6)
Absent		4 (3.0)	50 (37.9)	44 (33.3)	34 (25.8)	132 (61.4)

**Figure 1:**
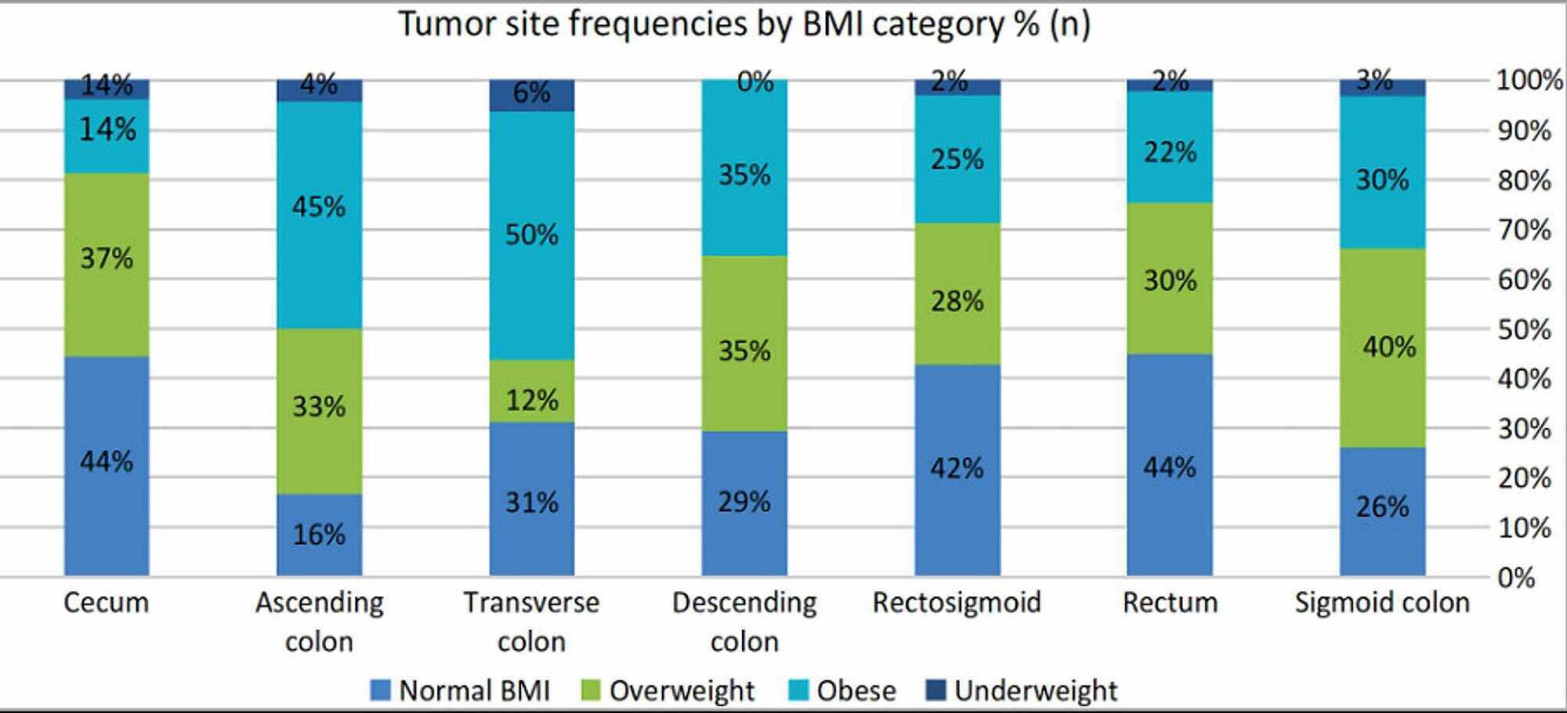
Tumor site frequencies by BMI category BMI, body mass index

**Figure 2:**
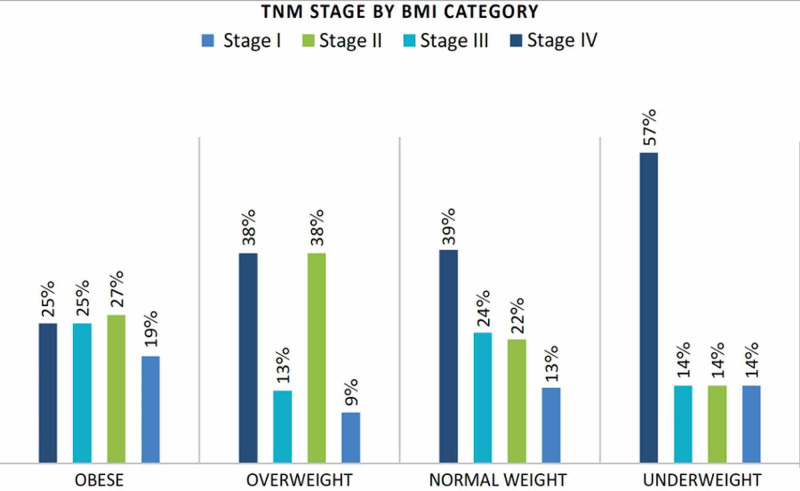
TNM stage by BMI category BMI, body mass index; TNM, tumor node metastasis

Of 16 patients with transverse colon cancer, 8 were obese (50%), and 1 (6%), 5 (31%), and 2 (13%) patients were underweight, normal weight, and overweight, respectively, as shown in Figure 1. The median lymph node yield was 14, and the values were relatively comparable among all BMI categories (Table 2). In total, 17 of 69 obese patients (25.8%) had Stage IV disease. Meanwhile, 57.1%, 39.2%, and 38.7% of underweight, normal, and overweight patients, respectively, had Stage IV disease. There were 128 (57%) patients with advanced tumor stage (III and IV) at the time of diagnosis. Of those patients, 81 (36%) had distant metastasis (Table 2, Figure 2). The tumor stage was not related to the BMI category in the bivariate analysis. Similarly, there was no statistically significant relationship between BMI category and the histopathological type, tumor location, degree of differentiation, lymph node yield, or the number of involved lymph nodes (N) (Table 2).

 

## Discussion

Although obesity is an established risk factor for CRC, the relationship between BMI and clinicopathologic characteristics (histological type, degree of differentiation, tumor location, and medical comorbidities) and prognostic factors (TNM stage, lymph nodes involvement, and lymph nodes yield) in patients with CRC remains unclear to date. In this study, we found no relationship between BMI category and the tumor stage. However, metastatic disease was more frequent in normal weight and overweight patients than in obese patients. This could be explained by different factors. First, rapid unintentional weight loss is expected in advanced-stage disease [12], making the certainty of the actual patient’s weight, and therefore BMI, questionable. Second, malignancy-related catabolic states, cachexia, and poor nutritional status of patients would ultimately negatively affect immune response that could consequently lead to further progression of the disease [17]. Finally, the proposed protective gene expressions and behavioral tumor features related to high BMI could be another explanation [18].

Our findings are consistent with those of other studies that showed a lack of significant relationship between BMI and tumor stage [12,19]; however, this relationship was clinically appreciable. In one study, no reported obese patient had advanced cancer stage [19]. These findings could, in part, be explained by the obesity paradox reported in several studies, in which overweight and obese patients had better survival rates than their normal and underweight counterparts [20]. In contrast, obesity has also been reported to be an adverse factor in patients with CRC, with obese patients presenting with advanced tumor stage at diagnosis [21]. A large prospective cohort study concluded that high BMI and other anthropometric measures were significantly associated with advanced-stage CRC in both sexes, with the association being more significant in males [22]. A meta-analysis of 29 studies concluded that the timing of BMI measurement was an important factor to be considered when taking into account the prognosis of patients with CRC and showed that pre-diagnostic obesity increased overall mortality, while post-diagnostic overweight improved survival [23]. These conflicting results are possible because of the complexity and diversity of the involved factors of CRC pathogenesis and its clinical assessment.

Lymph node yield is an important predictor of survival and patient outcomes. A systematic review found that high lymph node yield is associated with better survival [24]. In general, at least 12 lymph nodes are the minimum accepted yield of CRC surgery. In this study, the median lymph node yield was 14, and there was no significant difference in the lymph node yield among the BMI categories (p = 0.89). Importantly, there was no significant relationship between BMI category and the number of involved lymph nodes (p = 0.94). In contrast, some studies found a significantly higher number of involved lymph nodes in obese patients with colon cancer than in patients with normal BMI [25]. In addition, a 2017 meta-analysis showed a higher probability of lymph node metastasis in patients who are obese than in those in lower BMI categories [11].

In this study, the sigmoid colon was the most prevalent tumor site, accounting for 65 of 233 cases. Meanwhile, the least common site was the transverse colon (16/233). Surprisingly, there was no significant relationship between the tumor site and all BMI categories (p = 0.38). This is in contrast to the findings of a systematic review and meta-analysis that showed a relationship between high BMI and distal colon cancer; however, this relationship was not strong [26]. Tumor grade (degree of differentiation) has been shown to be an independent variable to predict the prognosis of colon cancer [27]. Leptin, a hormone that increases in response to increased body fat percentage [28], was found to be overexpressed in patients with moderately differentiated CRC and was found to be in low concentration or absent in patients with normal colonic tissues in one study [29]. These findings indicate that leptin may play a role in colorectal carcinogenesis; however, a causal relationship was still not determined. The majority (81.5%) of our patients had moderately differentiated CRC, and only 11.5% and 7% of patients had well and poorly differentiated tumors, respectively. Furthermore, we did not determine a relationship between tumor grade and BMI categories (p = 0.71). This finding is in line with the results of a meta-analysis that included 29 studies and found no significant difference in the CRC grade between obese and non-obese patients [11]. Meanwhile, a study conducted in Spain found that overweight and obese patients with colon cancer had more poorly differentiated tumors than their normal-weight counterparts. However, this relationship was not found in patients with rectal cancer [19]. The current evidence does not suggest the presence of a relationship between BMI and tumor grade.

The histopathological variants of CRC are closely associated with the rate of tumor growth and degree of differentiation and can thus be used to predict its prognosis. In the current study, the most prevalent histopathological type was adenocarcinoma (200/233 patients, 85.8%). Our results showed no relationship between BMI and histopathological types (p = 0.91).

This study had some limitations, including a small sample size. Additionally, the causation relationship between the variables were not identified owing to the descriptive design of the study. Despite these limitations, we believe that this study remains valuable because to the best of our knowledge, this is the first study to investigate the relationship of BMI with clinicopathological and prognostic factors of CRC in Saudi Arabia. Studies with larger sample sizes are needed to validate our findings. Future studies also need to measure body composition using waist circumference and/or the waist-to-hip ratio instead of BMI, given that central obesity is a stronger predictor for the risk of colon cancer [30]. Studies with a prospective design and larger sample sizes are also recommended to better establish the effect of BMI on CRC. Moreover, the prognostic effect of comorbidities on CRC should be investigated because the findings may provide useful data related to screening and early detection.

## Conclusions

Patients with transverse CRC are more likely to be obese, while underweight patients are more likely to present with Stage IV disease, indicating that patients in this BMI group may have the worst prognosis. However, BMI is not related to lymph node yield, histological type, or the degree of differentiation. These findings can be helpful for the development of a more individualized approach to patient management based on patients’ BMI. Future studies should measure body composition with tools that reflect visceral obesity more accurately than BMI to investigate the effect of body composition on CRC.

## References

[1] Kuipers EJ, Grady WM, Lieberman D (2015). Colorectal cancer. Nat Rev Dis Primers.

[2] Bazarbashi S, Al Eid H, Minguet J (2017). Cancer incidence in Saudi Arabia: 2012 data from the Saudi Cancer Registry. Asian Pac J Cancer Prev.

[3] Amersi F, Agustin M, Ko CY (2005). Colorectal cancer: epidemiology, risk factors, and health services. Clin Colon Rectal Surg.

[4] Ng M, Fleming T, Robinson M (2014). Global, regional, and national prevalence of overweight and obesity in children and adults during 1980-2013: a systematic analysis for the Global Burden of Disease Study 2013. Lancet.

[5] Goday A, Barneto I, García-Almeida JM (2015). Obesity as a risk factor in cancer: a national consensus of the Spanish Society for the Study of Obesity and the Spanish Society of Medical Oncology. Clin Transl Oncol.

[6] Bardou M, Barkun AN, Martel M (2013). Obesity and colorectal cancer. Gut.

[7] Renehan AG, Tyson M, Egger M (2008). Body-mass index and incidence of cancer: a systematic review and meta-analysis of prospective observational studies. Lancet.

[8] Hidayat K, Yang C-M, Shi B-M (2018). Body fatness at an early age and risk of colorectal cancer. Int J Cancer.

[9] Haydon AM, Macinnis RJ, English DR (2006). Effect of physical activity and body size on survival after diagnosis with colorectal cancer. Gut.

[10] Shaukat A, Dostal A, Menk J (2017). BMI is a risk factor for colorectal cancer mortality. Dig Dis Sci.

[11] Rogers AC, Handelman GS, Solon JG (2017). Meta-analysis of the clinicopathological characteristics and peri-operative outcomes of colorectal cancer in obese patients. Cancer Epidemiol.

[12] Walter V, Jansen L, Hoffmeister M (2016). Prognostic relevance of prediagnostic weight loss and overweight at diagnosis in patients with colorectal cancer. Am J Clin Nutr.

[13] Schlesinger S, Siegert S, Koch M (2014). Postdiagnosis body mass index and risk of mortality in colorectal cancer survivors: a prospective study and meta-analysis. Cancer Causes Control.

[14] Lu Y, Ness-Jensen E, Martling A (2016). Anthropometry-based obesity phenotypes and risk of colorectal adenocarcinoma: a large prospective cohort study in Norway. Epidemiology.

[15] Edge SB, Compton CC (2010). The American Joint Committee on Cancer: the 7th Edition of the AJCC Cancer Staging Manual and the Future of TNM. Ann Surg Oncol.

[16] (1995). Physical status: the use and interpretation of anthropometry. Report of a WHO Expert Committee. World Health Organ Tech Rep Ser.

[17] Thoresen L, Frykholm G, Lydersen S (2013). Nutritional status, cachexia and survival in patients with advanced colorectal carcinoma. Different assessment criteria for nutritional status provide unequal results. Clin Nutr.

[18] Brändstedt J, Wangefjord S, Borgquist S (2013). Influence of anthropometric factors on tumour biological characteristics of colorectal cancer in men and women: a cohort study. J Transl Med.

[19] García-Oria Serrano MJ, Armengol Carrasco M, Ortiz R (2010). The impact of obesity on the histopathological characteristics of colorectal tumours. An observational study. Cirugía Española.

[20] Azvolinsky A (2014). Cancer prognosis: role of BMI and fat tissue. J Natl Cancer Inst.

[21] Neumann K, Mahmud SM, McKay McKay (2015). Is obesity associated with advanced stage or grade of colon cancer? Canadian journal of surgery. Can J Surg.

[22] Brändstedt J, Wangefjord S, Nodin B (2012). Gender, anthropometric factors and risk of colorectal cancer with particular reference to tumour location and TNM stage: a cohort study. Biol Sex Differ.

[23] Wu S, Liu J, Wang X (2014). Association of obesity and overweight with overall survival in colorectal cancer patients: a meta-analysis of 29 studies. Cancer Causes Control.

[24] Chang GJ, Rodriguez-Bigas MA, Skibber JM (2007). Lymph node evaluation and survival after curative resection of colon cancer: systematic review. J Natl Cancer Inst.

[25] Meyerhardt JA, Catalano PJ, Haller DG (2003). Influence of body mass index on outcomes and treatment-related toxicity in patients with colon carcinoma. Cancer.

[26] Robsahm TE, Aagnes B, Hjartåker A (2013). Body mass index, physical activity, and colorectal cancer by anatomical subsites: a systematic review and meta-analysis of cohort studies. Eur J Cancer Prev.

[27] Greene FL, Stewart AK, Norton HJ (2002). A new TNM staging strategy for node-positive (stage III) colon cancer: an analysis of 50,042 patients. Ann Surg.

[28] Considine RV, Sinha MK, Heiman ML (1996). Serum immunoreactive-leptin concentrations in normal-weight and obese humans. N Engl J Med.

[29] Koda M, Sulkowska M, Kanczuga-Koda L (2007). Overexpression of the obesity hormone leptin in human colorectal cancer. J Clin Pathol.

[30] Moore LL, Bradlee ML, Singer MR (2004). BMI and waist circumference as predictors of lifetime colon cancer risk in Framingham Study adults. Int J Obes Relat Metab Disord.

